# Stem cell programs in cancer initiation, progression, and therapy resistance

**DOI:** 10.7150/thno.41648

**Published:** 2020-07-09

**Authors:** Tianzhi Huang, Xiao Song, Dandan Xu, Deanna Tiek, Anshika Goenka, Bingli Wu, Namratha Sastry, Bo Hu, Shi-Yuan Cheng

**Affiliations:** 1 The Ken & Ruth Davee Department of Neurology, Lou and Jean Malnati Brain Tumor Institute, The Robert H. Lurie Comprehensive Cancer Center, Northwestern University Feinberg School of Medicine, Chicago, IL 60611, USA; 2 Department of Pathology, Feinberg School of Medicine, Northwestern University, Chicago, IL 60611, USA

**Keywords:** cancer stem cells, transcriptional and posttranslational regulation, epigenetics, metabolism, tumor microenvironment, therapy resistance, CSC-targeting therapies

## Abstract

Over the past few decades, substantial evidence has convincingly revealed the existence of cancer stem cells (CSCs) as a minor subpopulation in cancers, contributing to an aberrantly high degree of cellular heterogeneity within the tumor. CSCs are functionally defined by their abilities of self-renewal and differentiation, often in response to cues from their microenvironment. Biological phenotypes of CSCs are regulated by the integrated transcriptional, post-transcriptional, metabolic, and epigenetic regulatory networks. CSCs contribute to tumor progression, therapeutic resistance, and disease recurrence through their sustained proliferation, invasion into normal tissue, promotion of angiogenesis, evasion of the immune system, and resistance to conventional anticancer therapies. Therefore, elucidation of the molecular mechanisms that drive cancer stem cell maintenance, plasticity, and therapeutic resistance will enhance our ability to improve the effectiveness of targeted therapies for CSCs. In this review, we highlight the key features and mechanisms that regulate CSC function in tumor initiation, progression, and therapy resistance. We discuss factors for CSC therapeutic resistance, such as quiescence, induction of epithelial-to-mesenchymal transition (EMT), and resistance to DNA damage-induced cell death. We evaluate therapeutic approaches for eliminating therapy-resistant CSC subpopulations, including anticancer drugs that target key CSC signaling pathways and cell surface markers, viral therapies, the awakening of quiescent CSCs, and immunotherapy. We also assess the impact of new technologies, such as single-cell sequencing and CRISPR-Cas9 screening, on the investigation of the biological properties of CSCs. Moreover, challenges remain to be addressed in the coming years, including experimental approaches for investigating CSCs and obstacles in therapeutic targeting of CSCs.

## Introduction

Tumors are complex systems that include cancerous cells as well as the associated tumor microenvironment (TME), which is comprised of cancer-associated fibroblasts (CAFs), vasculature, infiltrating immune cells, and other components. Considerable efforts have been made to model the complexity of cancer by integrating the TME to interrogate tumor heterogeneity based on epigenetic and genetic variations. The concept of cancer stem cells (CSCs) states that tumors, like their normal tissue counterparts, have a unidirectional cellular hierarchy with CSCs at the apex, which are responsible for sustaining tumorigenicity and recapitulating the cellular heterogeneity inherent within the original tumor 1. The CSC model stimulated intense interest when evidence emerged supporting the model in human acute myeloid leukemia (AML) and breast cancer. It was found that a small subpopulation of cancer cells were capable of initiating leukemia when transplanted into immune-deficient mice. Moreover, these AML cells could be enriched by using a combination of the cell surface markers CD34^+^CD38^-^ 2, while breast cancer-propagating cells were marked with CD44^+^CD24^-/low^ 3. There is now convincing evidence for CSCs in a variety of solid tumors, including brain 4, 5, prostate 6, 7, colon 8, 9, pancreatic 10, ovarian 11, and lung 12. Although the existence of CSCs has been evident in various cancer types, it remains uncertain whether the CSC model applies to all or only some cancers due to issues concerning the robustness of CSC markers and the extent to which existing assays underestimate the tumorigenic cell frequencies.

The CSC model has received wide attention because it explains the clinical observation that many treatments seem to initially eradicate cancer cells, but later the cancer will often return. Therefore, it is of considerable importance in the clinic to target CSCs within the tumor to prevent tumor relapse. CSCs are often more resistant to currently available anticancer therapies than the rapidly dividing bulk tumor cells surrounding the CSC subpopulation. Sporadic genetic mutations and epigenetic changes within the tumor cells, as well as interactions with the TME, influence both CSCs and the tumors response to therapies and overall prognosis 13, 14. In the following sections, we provide an update on intrinsic and extrinsic regulators of CSCs, including transcriptional and post-transcriptional regulation, epigenetics, metabolism, and the TME that have shaped our understanding of how CSCs function to drive tumor growth and therapeutic resistance. We also highlight recent developments of CSC-based therapies and clinical trials.

## CSC definition and key features

Various methods have been developed to characterize CSCs and determine the degree to which a cell possesses the ability to self-renew 15. CSCs form spheres *in vitro* that are maintained through serial passages, while progenitor or differentiated cells lack this ability 16. Moreover, unlike differentiated cells, *in vivo* xenografts with CSCs yield sizable tumors in immunocompromised mice, and these can be faithfully recapitulated with serial transplantations. In addition, cell surface markers have been a useful tool to characterize CSCs, as many of these markers are present on CSCs and normal stem cells but are not expressed on differentiated tumor cells 17. For instance, CD133 is a marker for hematopoietic stem cells (HSCs), but has been widely acknowledged as a CSC marker in breast, prostate, colon, glioma, liver, lung, and ovarian cancers. Finally, lineage tracing studies are able to use markers (e.g. GFP) to monitor the ability of a cell that gives rise to and maintains clonal progeny containing the parental marker 1. CSCs that can grow and maintain these colonies demonstrate a hierarchical organization structure.

There is growing evidence indicating that a tumor mass composed of CSCs, differentiated cancer cells, and the non-malignant stromal cell network all work together to allow the tumor to adapt and thrive in the harsh TME 18. A well-characterized example of cellular plasticity in normal cells is the intestinal stem cell population 19, in which certain differentiated endocrine cells modulate their genetic profiles to resemble intestinal stem cells after tissue injury 20. Moreover, in colorectal cancer with genetic ablation of Leucine Rich Repeat Containing G Protein-Coupled Receptor 5^+^ (LGR5^+^) CSCs, differentiated keratin 20^+^ (KRT20^+^) cancer cells become dedifferentiated upon entering the niche previously occupied by the ablated LRG5^+^ CSCs 21. Such functional plasticity is also seen in glioma stem-like cells (GSCs). Upon treatment with receptor tyrosine kinase (RTK) inhibitors, GSCs can adopt a slow cell cycling state that is dependent upon Notch signaling and is associated with chromatin remodeling using H3K27 demethylases 22. This epigenetic modulation allows GSCs to persist when confronted with therapeutic insults, thereby providing an avenue for therapeutic resistance. In breast cancer, differentiated basal and luminal cells can revert to a stem cell-like state at a low but significant rate 23. Given sufficient time, subpopulations of stem, basal, or luminal cells cultured individually can eventually recapitulate phenotypic proportions that include the other two cell types, thereby mirroring the heterogeneity of clinical breast cancer. The ability of cancer cells to endure therapeutic stress is once again evident in this situation 1, 23. Unlike stem cells, basal and luminal breast cancer cells are normally unable to give rise to tumors in mice. However, upon co-inoculation with irradiated cells, all three subpopulations are effectively tumorigenic.

## The transcriptional regulation of CSCs

CSCs have the ability to self-renew and differentiate which allows them to not only be tumorigenic, but also possess the plasticity to promote drug/radiation resistance following treatment. These processes involve multiple critical and highly regulated transcription factors (TFs), which govern CSC homeostasis. CSCs also express several critical TFs that play a key role in inducing pluripotency in somatic cells, including octamer-binding transcription factor 4 (OCT4), Sry-related HMG box 2 (SOX2), Kruppel-like factor 4 (KLF4), NANOG, and c-MYC 24-26. In addition, many intracellular signaling pathways 27, such as Wnt/TCF, Signal transducer and activator of transcription 3 (STAT3), and NF-κB also have important roles in the regulation of CSC phenotypes (Table 1). In particular, the central stemness-associated TFs, OCT4, SOX2, KLF4, c-MYC, and NANOG are expressed in both CSCs and normal stem cells, such as embryonic stem cells (ESCs) 28. Accumulating evidence shows that the overexpression of these central stemness-associated TFs occurs in various types of human cancers, including breast cancer 29, prostate cancer 30, and oral squamous cell carcinoma 31. The aberrant expression of these TFs is associated with tumor initiation, progression 29, and therapy resistance 32. The central stemness-associated TFs play critical roles in maintaining the pluripotency and self-renewal properties of CSCs and ESCs, but with distinct mechanistic functions between them 28. Although CSCs and ESCs share common properties such as self-renewal, they have different phenotypic features and proliferative potentials 33. The central stemness-associated TFs play a vital role in the maintenance of self-renewal and pluripotency in ESCs as well as in the control of early cell fate decisions in ESCs 34. However, the overexpression of these central stemness-associated TFs in CSCs regulates signaling pathways to promote tumorigenicity and cell survival in response to cancer treatments 28.

## OCT4

OCT4, encoded by the *POU5F1* gene, is implicated in multiple processes, including stem cell maintenance and embryogenesis 35. OCT4 is also overexpressed in CSCs of various types of human cancers, and has a positive association with tumorigenesis, therapy resistance, and a worse prognosis in cancers 36, 37. Suppression of OCT4 increases sensitivity to irradiation and chemotherapy in lung cancer 38, glioma 39, and oral squamous cancer cells 40. In pancreatic CSCs (PCSCs), combined inhibition of OCT4 and NANOG suppressed the proliferation, migration, invasion of CSCs *in vitro*, and the tumorigenicity of CSCs in immunocompromised mice, but increased the chemosensitivity of CSCs 41. OCT4-regulated expression of EMT-related genes, *CXCR4*, *MMP2*, *MMP9,* and *TIMP1* is associated with tumor growth, metastasis, and drug resistance of PCSCs 41. Mouse breast cancer cells with elevated expression of OCT4 have an increased ability of forming tumor spheres, and a worse high levels of stemness-associated genes such as *ATXN1*, *PROM1*, *CD34*, and *ALDH1*, thereby displaying higher tumorigenic potential *in vivo* 42.

## SOX2

SOX2 plays an essential role in embryonic development and the maintenance of stemness in embryonic and adult stem cells 43. Dysregulated expression of SOX2 is associated with cancer pathogenesis and several traits of cancer cells such as proliferation, EMT, CSC formation, resistance to apoptosis, and chemotherapy 44. Moreover, accumulating evidence shows that SOX2 is involved in the regulation of self-renewal, tumor growth, and therapy resistance of CSCs in cancer 44, 45. In glioblastoma (GBM), suppression of SOX2 in GSCs resulted in cell cycle arrest and markedly reduced their abilities of cell growth, migration, invasion, and tumorigenicity 46. In breast cancer, SOX2 is required for the proper functioning of CSCs, and mediates resistance to the estrogen receptor antagonist tamoxifen 47, 48. Given the roles of SOX2 in cancer biology and CSCs, it is of high importance to investigate the downstream regulatory pathways either directly or indirectly mediated by SOX2 and the development of specific SOX2-targeting cancer therapies.

## KLF4

KLF4 is a zinc finger transcription factor that is involved in diverse cellular processes, including regulation of the cell cycle and maintenance of cellular pluripotency 49. Since the discovery of KLF4 in 2006 as one of four key factors required for the induction of pluripotent stem cells (iPSCs) 24, there has been an increase in research to determine the function of KLF4. MicroRNA-7 (miR-7) suppresses the expression of KLF4 by directly targeting its 3'-untranslated region, which leads to a decrease in brain metastasis 50 of breast CSCs. In glioma, KLF4 was found to directly bind to the promoter region of *Integrin β4 (ITGB4)* and increase *ITGB4* expression, resulting in an increase in GSC self-renewal and enhanced glioma cell migration and proliferation both *in vitro* and *in vivo* 51. Furthermore, KLF4 was shown to enhance the sphere-formation ability, drug resistance, and metastatic potential in osteosarcoma CSCs through activation of the p38 mitogen-activated protein kinase (MAPK) signaling pathway 52. Taken together, these studies reveal an essential role for KLF4 in the regulation of CSC properties and suggest KLF4 as a potential therapeutic target for cancer treatments.

## c-MYC

MYC proteins, including c-MYC, N-MYC, and L-MYC have important roles in tumorigenesis and therapeutic resistance. Among these MYC members, c-MYC is perhaps the most frequently dysregulated protein in human tumorigenesis, thereby serving as a promising therapeutic target for cancer treatments 53. c-MYC amplification in patient-derived GSCs generates sensitivity to PARP inhibition via c-MYC-mediated transcriptional repression of *CDK18*, *CCNE2*, and *CDKN1A* 54. Similarly, in triple-negative breast cancer, c-MYC interacts with a lncRNA, *LINC01638,* which prevent its ubiquitination and degradation. In turn, c-MYC transcriptionally regulates *MTDH* expression and then stimulates Twist1 signaling to maintain an EMT signature and CSC-like state 55.

## NANOG

As one of the central stemness-associated TFs, NANOG plays a critical role in embryonic development and cellular reprogramming 24. NANOG is broadly expressed in various types of cancers 56. Recently, the involvement of NANOG in tumorigenesis and cancer progression has drawn significant attention. Aberrant overexpression of NANOG has been described in hepatic, prostate, colorectal, and brain CSCs 4, 57-59. Correlative expression between NANOG and CSC markers has also been reported. For example, NANOG is enriched in CD133^+^ or CD44^+^ cancer cells as compared to CD133^-^ or CD44^-^ cells 60-62. NANOG regulates the fundamental properties of CSCs such as cell proliferation, cell cycle, self-renewal, EMT, tumorigenicity, and chemoresistance 56, 62, 63. Therefore, NANOG represents a critical molecular nexus underlying tumor initiation and progression and may prove to be a novel therapeutic target for CSC elimination.

## Wnt/TCF4

Wnt signaling is one of the key cascades in control of embryonic development and is also involved in self-renewal, tumorigenesis, and metastasis of CSCs 64. Several components in the Wnt signaling pathway, such as LEF1, cyclin D1, β-catenin, and TCF-4 are expressed at markedly higher levels in the CSC population as compared to non-CSCs 65. The decreased Wnt1 in breast CSCs caused the down-regulation of the stemness-associated genes *CD44*, *ALDH1*, and *ATXN1*, as well as reductions in the CSC subpopulation and tumor sphere formation 66. Additionally, Wnt signaling activity is enriched in the proneural subtype of GSCs. Wnt signaling is tightly associated with CSC properties through the induction of miR-20b and miR-125b transcription: these miRs function as suppressors of two negative regulators of Wnt signaling, FZD6 and APC 67.

## STAT3

STAT3 is dysregulated in a number of human cancers, acting as a key molecular driver in multiple signaling processes. Phosphorylated STAT3 at tyrosine (Tyr) 705 by Janus kinases (JAK), regulates its dimerization, nuclear accumulation, and DNA binding, thereby initiating its transcriptional regulation 68. STAT3 is centrally important for the maintenance of CD44^+^/CD24^-^ CSCs in breast cancer, suppression of genes involved in cell proliferation correspondingly reduce STAT3 activation 69. Furthermore, in colon cancer, the internalized CD44 and acetyltransferase p300 induce STAT3 acetylation at Lysine (Lys) 685, which in turn promotes the expression of cell cycle regulators cyclin D1 70, MYC, and Twist1 in colon cancer cells 71. Moreover, in hepatocellular carcinoma, activated STAT3 can increase CD133 expression through functional cooperation with NF-κB and hypoxia inducible factor 1 alpha (HIF-1α) 72. STAT3 is also a key regulator of GSCs. STAT3 inhibition suppresses the expression of stemness-associated genes *OLIG2* and *NES*, but enhances expression of the differentiation marker *TUBB3* 73. Additionally in leukemia, inhibition of STAT3 with its inhibitor AZD9150 leads to deceased expression of leukemic drivers including *IL1RAP*, *MSI2*, *CXCR2,* and *IL8*, as well as *MCL1* 74. Similarly, the inactivation of STAT3 in *PTEN*-deficient tumors activates immunosurveillance in prostate cancer by reducing the expression of cytokines including CXCL2, GM-CSF, M-CSF, C5a, IL10, and IL13, thereby displaying the immunostimulatory features of the senescence-associated secretory phenotype of tumor cells 75.

## NF-κB

NF-κB is critical in the toll-like receptor 2 (TLR2)- initiated TLR2-MyD88-NF-κB signaling pathway, which creates a pro-inflammatory microenvironment and supports self-renewal of epithelial ovarian CSCs, through up-regulation of CD44, NANOG, SOX2, as well as IL-6 76. NF-κB co-operates with β-catenin to enhance the expression of *CD44*, *ALDH1*, *MYC*, and *OCT4,* known markers of breast CSCs, which results in an enriched CD44^high^/CD24^-/low^ sub-population post poly(I:C) treatment 77. On the other hand, NF-κB plays a critical role in the expansion of breast CSCs through heterotypic signals that promote macrophage recruitment and angiogenesis 78. TLR9 is essential for the propagating potential of prostate cancer cells via activation of NF-κB and STAT3, which induces the expression of key stem cell-related genes, including *NKX3.1*, *KLF-4*, *BMI-1*, and *COL1A1* by directly interacting with their promoters 79. In PCSCs, NF-κB induces SOX9 expression, which is critical in controlling the CSC population and invasion ability of the pancreatic cancer cells 80. Finally in GSCs, the STAT3/NF-κB signaling pathway is constitutively activated in both adherent and spheroid GSCs, and helps to up-regulate the positive regulators of the Notch pathway, including NOTCH1, NOTCH3, NOTCH4, HES5, HEY1, and JAG1, but also down-regulates the negative regulators, such as CTBP1 and RBPJ 81.

## Post-transcriptional regulation in CSCs

Advancements in technology and methodology have broadened our understanding of post-transcriptional modifications as an additional regulatory mechanism of gene expression in cancer. The term “epitranscriptome” 82 was coined to refer to diverse post-transcriptional modifications on RNA, including various RNA processing events such as RNA methylation, editing, and alternative splicing. Emerging data suggest that epitranscriptome regulation plays a central role in CSC maintenance and cancer development 83.

## RNA methylation in CSCs

**Figure 1:**
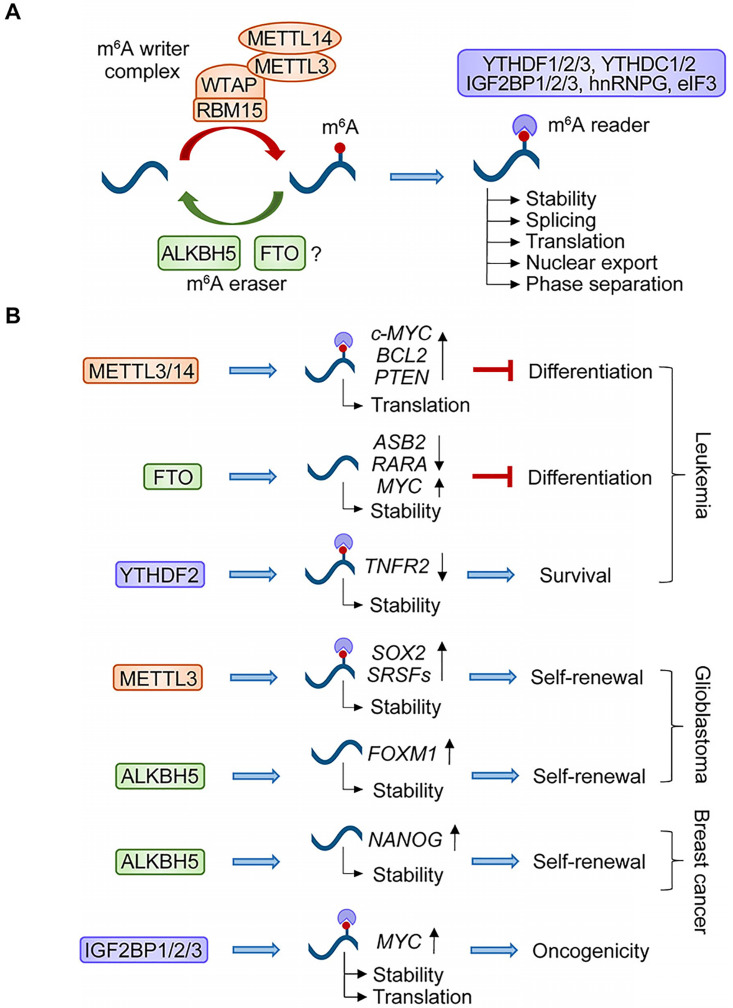
** Mechanism of N^6^-methyladenosine (m^6^A) modification and its roles in CSCs. (A)** m^6^A is regulated by writers, erasers, and readers. “Writers” refer to the m^6^A methyltransferase complex including METTL3, METTL14, WTAP, and RMB15. “Erasers” are m^6^A demethylases including ALKBH5 and FTO. “Readers” are proteins that recognize m^6^A, including YTH domain containing proteins (YTHDF1/2/3 and YTHDC1/2), IGF2BP1/2/3, and other factors such as hnRNPG and eIF3. The binding of these “readers” to m^6^A mediates downstream RNA processes, including stability, splicing, nuclear export, translation, and phase separation potential of targeted mRNAs. **(B)** The m^6^A modification and its regulators play critical roles in cancer stem cell maintenance and tumorigenicity.

N^6^-methyladenosine (m^6^A) represents the most widely distributed internal mRNA modification in mammals, with around 25% of mRNAs containing at least one m^6^A site 84. As a dynamic process, m^6^A is catalyzed by RNA methyltransferases known as “writers” and can be removed by RNA demethylases called “erasers” (Figure 1A). A number of proteins have been identified in the m^6^A writer complex, including methyltransferase-like 3 (METTL3, catalytic component), Wilms tumor 1-associated protein (WTAP, key adaptor for METTL3), methyltransferase-like 14 (METTL14, RNA adaptor needed for METTL3 activity), RNA binding motif protein 15 (RBM15, mediator of methylation specificity through binding to U-rich regions in mRNAs), and Vir Like M^6^A Methyltransferase Associated (VIRMA) 85. Two different enzymes were identified as m^6^A demethylases: fat mass and obesity-associated protein (FTO) and alkylated DNA repair protein AlkB homolog 5 (ALKBH5). However, recent studies using methylated RNA immunoprecipitation sequencing (MeRIP-Seq), and several other findings have shown that FTO physiologically targets m^6^A_m_ (N^6^,2′-O-dimethyladenosine), which is indistinguishable from m^6^A, suggesting that m^6^A might not be the correct substrate of FTO 86. Additionally, several m^6^A-binding proteins, such as YTH domain containing protein family (YTHDF1/2/3 and YTHDC1/2) and insulin-like growth factor 2 mRNA-binding proteins (IGF2BP1/2/3), have been identified as m^6^A “readers”. By recruiting various “readers”, m^6^A exerts diverse effects on RNA metabolism, stability (YTHDF2), splicing (YTHDC1 and hnRNPG), nuclear export (YTHDC1), translation efficiency (YTHDF1, IGF2BPs, eukaryotic initiation factor 3, eIF3), and phase separation potential (YTHDF1/2/3) of targeted mRNAs 87-93.

Given the critical role of m^6^A modifications in regulating cellular pluripotency and differentiation 94, their linkage with CSC maintenance and tumorigenesis is not surprising. Studies strongly support the critical requirement for the m^6^A “writer” complex in AML (Figure 1B). Depletion of METTL3, METTL14, or WTAP results in cell-cycle arrest, differentiation of leukemic cells, and delayed leukemogenesis *in vivo*, through m^6^A modifications of target mRNAs, such as c-*MYC*, *BCL2*, *PTEN*, *SP1*, *SP2,* and *MYB* 95-97. FTO also exerts an oncogenic role in AMLs, which suppresses leukemia cell differentiation and enhances leukemogenesis by modulating the expression of targets such as *ASB2*, *RARA*, and* MYC* 98, 99. Targeting YTHDF2 stabilizes m^6^A-modified transcripts, such as *TNFR2*, and selectively compromises AML development without dampening normal hematopoiesis 100. In glioma (Figure 1B), studies addressing the roles of m^6^A in GSCs have generated divergent results. For example, treatment of serum and retinoic acid elevated the level of m^6^A in GSCs, inducing GSC differentiation, while knockdown of METTL3 or METTL14 promoted growth, self-renewal, and tumorigenesis 101. Controversially, other studies showed that global m^6^A levels were decreased during serum-induced GSC differentiation and METTL3-mediated m^6^A modifications play essential roles in the maintenance and tumorigenicity of GSCs through stabilizing *SOX2* and *SRSF* transcripts 102, 103. ALKBH5 is overexpressed in GSCs and plays a critical role in GSC self-renewal and tumorigenesis through demethylating *FOXM1* nascent transcripts and enhancing FOXM1 expression 104. In breast cancer (Figure 1B), hypoxia-induced ALKBH5 expression in breast cancer cells demethylated and stabilized *NANOG* mRNA, thereby promoting the self-renewal of breast CSCs 105. IGF2BPs play oncogenic roles in cancers as m^6^A readers by enhancing mRNA stability and translation of *MYC* transcripts 92. Taken together, RNA methylation has been recognized as a critical regulator of CSC generation and maintenance by modulating the expression of various oncoproteins.

## RNA editing in CSCs

Comprehensive RNA sequencing has revealed extensive post-transcriptional modifications in the human transcriptome with the most prevalent form being adenosine-to-inosine (A-to-I) conversion 106. These modifications are catalyzed by adenosine deaminases acting on double-stranded RNA (ADAR) enzymes that include three ADAR members, ADAR1, ADAR2, and ADAR3. Following deamination of adenosine to inosine, the residue is interpreted as a guanosine (G) by the splicing and translational machinery, producing various functional results, such as changes in translated amino acids, biosynthesis and target recognition by small noncoding RNA, alternative RNA splicing, and lncRNA functions 107.

Although the overall biological functions of ADARs are still under investigation, aberrant activity of ADAR and dysregulated A-to-I RNA editing has been found in many human cancers 107, 108. In various tumor tissues, the level of A-to-I RNA editing is increased, probably due to the upregulation of ADAR1 in tumors 108. Several A-to-I RNA editing events have been found to correlate with cancer development and progression. In human chronic myeloid leukemia (CML), ADAR1 activation enhances self-renewal capacity of leukemia stem cells (LSCs) by editing *let-7* pri-microRNA (pri-miRNA), resulting in impaired *let-7* biogenesis and increased *LIN28B* pluripotency gene expression 109, 110. In hepatocellular carcinoma (HCC), ADAR1-induced amino acid substitution in antizyme inhibitor 1 (AZIN1) enhances its activity and promotes tumor initiation and development 111. Moreover, *SLC22A3* RNA editing is required for early tumor invasion and cell migration in familial esophageal cancer 112. In oral squamous cell carcinoma, ADAR1 promotes the EMT and stem-like cell phenotype by facilitating oncogenic onco-miRNA maturation. A systematic multi-cancer miRNA analysis identified A-to-I editing in miR-200b, where increased levels were found associated with a worse prognosis for cancer patients 113. Mechanistically, the edited miR-200b switches its role from suppressing to enhancing tumor invasion through targeting *LIFR*, an established inhibitor of cancer metastasis 113. ADAR2 is an essential enzyme for brain development 114 and is downregulated in gliomas 115. Overexpression of ADAR2 inhibits glioma cell proliferation and tumor growth by editing and reducing the expression of several onco-miRNAs, including miR-222/221 and miR-21 116, 117. Taken together, these studies suggest that aberrant regulation of RNA editing significantly contributes to the malignant phenotypes of CSCs.

## Dysregulation of RNA splicing in CSCs

Comprehensive transcriptomic analyses across cancer types have revealed widespread RNA alternative splicing (AS) alterations in tumors, which can be derived from somatic mutations in splicing-related genes, altered expression or activity of splicing factors, or mutations in cis-regulatory elements 118, 119. As an important regulator of embryonic stem cell pluripotency and reprogramming 120, RNA splicing also contributes to CSC generation and maintenance. For example, specific isoform expression signatures distinguish LSCs from normal human HSCs where the mis-spliced gene products include a pro-survival isoform of BCL-XL and splice variants of SHP-1 and PTK2B, which have been associated with hematological malignancies. More importantly, treatment with a spliceosome modulatory drug, 17S-FD-895, impairs LSC maintenance while sparing normal hematopoietic cells in humanized pre-clinical models 121. Moreover, specific splice variants of different stem cell regulatory RNAs, including *CD44v3* and exon 8, 9-deleted *GSK3β* enhances LSC self-renewal 122, 123. Protein arginine methyltransferase 5 (PRMT5) regulates the alternative splicing of genes involved in DNA repair, which maintain the genomic integrity of HSCs 124. Mutations affecting key spliceosome components, frequently occurring in hematologic malignancies, contribute to HSC maintenance and tumorigenic potential 125. Loss of SETD2/H3K36me3 mediates splicing modulation, regulates intestinal self-renewal, and aggravates Wnt/β-catenin-dependent colorectal tumorigenesis 126. The splicing factor, SRSF3, governs alternative splicing programs and promotes the self-renewal and tumorigenicity of GSCs 127. CD44 splice isoform switching was also shown to modulate the plasticity of breast CSC 128.

Together, recent studies revealed widespread alterations of RNA processing events in various types of human cancers. However, the events that have been functionally demonstrated as relevant to tumorigenesis or tumor development are still limited. Future studies should elucidate the extent to which epitranscriptomic alterations detected by next-generation sequencing technology can be incorporated into functionally relevant gene products and contribute to tumor development and malignancy. Moreover, other RNA processing events, such as alternative cleavage and polyadenylation, have been demonstrated to control cell fate decisions and pluripotency in iPSCs 129, but their involvement in CSC generation and maintenance remains largely unknown. Future studies aimed at comprehensively deciphering the epitranscriptome in CSCs and the crosstalk between different RNA processing events should unveil novel biomarkers and therapeutic targets in CSCs.

## CSC epigenetics

Tumor development and progression is regulated by both genetic and epigenetic modifications. The complex phases of tumorigenesis require discrete genetic changes in neoplastic cells and epigenetic alterations. Epigenetic changes within a cell do not involve primary DNA sequence alterations but rather accessibility of genetic loci to transcriptional machinery and chromatin remodeling, thus modulating DNA accessibility and transcription. Epigenetic mechanisms such as changes in DNA methylation, histone modifications, as well as noncoding RNAs, have been shown to play critical roles in cancer progression which influence cellular states at multiple steps in carcinogenesis. In the initial stage of cancer, changes in DNA methylation and chromatin caused by genetic mutations lead to oncogenic cellular reprogramming and acquisition of undifferentiated phenotypes 130. Convincing evidence in support of this view may come from findings in GBM, a lethal form of primary brain tumor characterized by high genetic heterogeneity. H3K27M mutations, which are found in over 70% of diffuse intrinsic pontine gliomas (DIPG), lead to a global reduction of the repressive H3K27me3 mark induced by the polycomb repressive complex 2 (PRC2), and drive neoplastic transformation in neural precursor cells and the induction of stemness properties 131, 132. As cancers grow and progress, additional epigenetic changes triggered by cell-extrinsic signaling from the TME affect behaviors and features of cells and help to establish tumor architecture 130.

## DNA methylation in CSCs

The complex phases of tumorigenesis cannot only be accounted for by discrete genetic alterations in neoplastic cells alone, but also involve epigenetic alterations. Proteins implicated in the regulation of DNA methylation are critical regulators of oncogenic self-renewal capacity. DNA methyltransferases, including DNMT1, DNMT3A, and DNMT3B, transfer a methyl-group to cytosines followed by guanine residues (CpG), while methylcytosine dioxygenases (TET1 and TET2) initiate a demethylation process by converting 5-methylcytosine (5mC) to 5-hydroxymethylcytosine. DNMT3A is a commonly mutated gene in ∼20% of patients with AML 133, resulting in inhibition of the methyltransferase activity of DNMT3A and expansion of preleukemic HSCs. Mutations in the TET proteins suppress the function of DNMTs 134. Furthermore, isocitrate dehydrogenase (IDH) is a gene that encodes the protein responsible for converting isocitrate to α-ketoglutarate, a required co-factor for TET and other dioxygenases 135. Interestingly, mutations in both TET and IDH proteins are associated with the onset and progression of myeloid malignancy. These data indicate that dysregulation of DNA methylation resulting from different genetic mutations can have similar results 133. In addition to leukemia, *IDH* mutations also frequently occur in gliomas. *IDH1* and/or *IDH2* mutations have been observed in a majority of patients with low-grade primary gliomas and secondary high-grade gliomas, which generate a genome-wide hypermethylation of CpG islands, known as the glioma-CpG island methylator phenotype (G-CIMP) 136. *IDH* mutations can reprogram committed cells and promote the acquisition of self-renewal capabilities. In normal human astrocytes, IDH1^R132H^ overexpression can increase cell proliferation and promote acquisition of stem cell characteristics 137. In addition to the canonical DNA methylation modification (5mC), noncanonical DNA methylation events occur on the sixth position of adenine bases (N6-methyladenine, N6-mA) where regulation by the DNA demethylase ALKBH1 plays an important role in GSC growth, self-renewal, and tumor formation capacity 138.

## Chromatin remodeling in CSCs

Chromatin remodelers and chromatin marks are frequently altered in human tumors. In AML CSCs, genes implicated in stemness maintenance, proliferation, or metabolism are marked with both H3K4me3 and H3K27me3. Moreover, during differentiation from CSCs to progenitor cells, genes related to stem cell identity were repressed via loss of the H3K4me3 mark alone 139. Similarly, DIPGs with H3K27M display a global reduction of H3K27me3, with specific enrichment at the loci of tumor suppressor genes. In these tumors, H3K27M binds to the catalytic site of the PRC2 and inhibits its methyltransferase activity 131, 140. Gene mutations in the subunits of PRC2 are frequently observed in cancer. Elevated expression of EZH2 is found to be associated with poor clinical prognosis and tumor invasiveness in various types of cancers 141. In addition, increased EZH2 expression is also associated with tumor progression and poor prognosis of glioma, wherein both genetic and pharmacological EZH2 inhibition eradicate self-renewal and tumorigenicity of GSCs 131, 142, 143. Additionally, EZH2 loss in combination with JAK2 V617F, RUNX1, TET2, or NRas G12D mutations can initiate myeloid or lymphoid malignancies 144-148. Both gain- and loss-of-function PRC2 mutations can be tumorigenic 149, 150, indicating that chromatin remodeling factors drive cancer initiation and progression in a context-dependent manner. Moreover, expression of EZH2 is not correlated with the abundance of H3K27me3 across breast cancer subtypes 151. Therefore, it is important to consider several aspects of chromatin remodelers in future studies, including the expression of chromatin remodelers to evaluate chromatin state, histone marks as a consequence of epigenetic regulators, and the undefined functions of chromatin remodelers beyond chromatin 151.

## Noncoding RNAs in CSCs

Noncoding RNA such as long noncoding RNAs (lncRNAs) and miRNAs play important roles in epigenetic modulations. LncRNA of transcription factor 7 (lncTCF7), which is overexpressed in liver CSCs, promotes self-renewal and tumor propagation of human liver CSCs through activation of Wnt signaling. This results from the recruitment of the Switch/sucrose nonfermentable (SWI/SNF) complex to the promoter of TCF7 which enhances TCF7 expression 152. Furthermore in glioma, epidermal growth factor receptor (EGFR) signaling-regulated lncRNA *NEAT1* regulates the Wnt/β-Catenin pathway by scaffolding EZH2 153. Moreover, LINC00339 is upregulated in glioma tumors and cells, and promotes cell proliferation, migration, invasion, and vascular mimicry formation by modulating the miR-539-5p/TWIST1/MMPs axis 154. Finally, lncRNA-Low Expression in Tumor (lncRNA-LET) participates in the development of chemo-resistance in urinary bladder cancers 155.

Over 2,500 miRNAs have been identified in humans, forming complicated regulatory networks in which each miRNA can simultaneously control the expression of various genes based on sequence homology, while each mRNA can be regulated by various miRNAs. Malignancy and stemness-associated miRNAs have been identified in GSCs, and their dysregulation is related with a poor prognosis of glioma patients, as well as cancer initiation and therapeutic resistance of CSCs 67, 156. As cancer is a disease with multiple gene and miRNA aberrations, miRNA-based strategies have the potential to be employed in combination with conventional therapies for cancer treatment 157. Overall, recent advances in epigenetics offer a better understanding of the epigenetic alterations underlying malignant transformation and provide novel opportunities for therapeutic strategies to target CSCs.

## CSC metabolism

Metabolic plasticity is a hallmark of cancer 158. Most cancer cells and other rapidly proliferating cells predominantly produce their energy through glycolysis followed by lactic acid fermentation, even in the presence of sufficient oxygen, a phenomenon termed the Warburg effect. Such aerobic glycolysis generates ATP less efficiently but more rapidly, and provides the building blocks for macromolecule synthesis, maintains redox homeostasis, and generates a tumor-supporting acidic microenvironment 159. Unlike bulk tumor cells which depend on glycolysis, CSCs demonstrate a unique metabolic flexibility. Several reports suggest that CSCs are primarily glycolytic and exhibit decreased mitochondrial function 160-165, whereas other studies, especially examination of patient-derived low-passage CSCs and chemoresistant CSCs, report an increased dependence on mitochondrial function and oxidative phosphorylation 166-173. Possible explanations for these discrepancies may be related to tumor types, environmental stimuli in the experimental system, and dynamic cellular phenotypes, including the transition from quiescent to proliferative CSCs. Several studies show that CSCs can switch between glycolysis and oxidative phosphorylation to survive in malleable, sometimes hostile environments such as hypoxia, starvation, or metastatic sites 167, 174-176.

In addition to the glucose-related metabolic reprogramming, lipid metabolism also regulates the functionality of CSCs in terms of self-renewal and tumorigenic abilities by building the plasma membranes, supplying bioenergy through fatty acid oxidation, and activating signal pathways as second messengers 160, 177-184. Additionally, increased amino acid metabolism, especially glutamine, fuels oxidative phosphorylation and favors survival in LSCs 185. Metabolism of lysine, serine, and branched-chain amino acids may also support CSC features 186-188. GSCs upregulate *de novo* purine synthesis under the transcriptional control of MYC, which maintains self-renewal, proliferation, and glioma sphere forming capacity 189.

Apart from providing substrates for cellular biosynthesis, metabolites could also actively affect stemness and lineage differentiation through epigenetic modulation. Histone acetylation is under the control of processes which influence the local acetyl-CoA pools, such as hypoxia, nutrient limitation, and intracellular acidification 190-192. The balance of DNA and histone methylation relies on the dynamics of methyl mark deposition and removal, under the control of S-adenosylmethionine metabolism and α-ketoglutarate/D-2-hydroxyglutarate metabolites that provide methyl groups and control the activity of demethylase enzymes, respectively 193. Small-molecule perturbation of the S-adenosylmethionine metabolism impacts the tumorigenicity of CSCs 194. Cancer-associated *IDH* mutations generate the oncometabolite, D-2-hydroxyglutarate, a competitive suppressor of α-ketoglutarate-dependent dioxygenases, and lock *IDH*-mutant cancer cells in a stem-like state 195-197.

## CSC microenvironment

**Figure 2:**
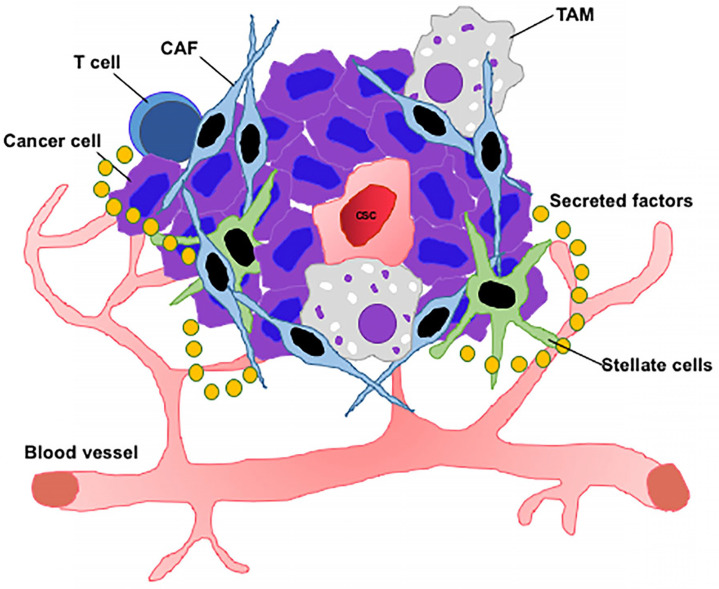
The TME consists of a heterogeneous population of cells including CSCs, dormant cancer cells, TAMs, T cells, other immune cells, and various secretory factors such as cytokines and growth factors.

The TME constitutes a heterogeneous population of neoplastic and non-transformed cells driven by both extrinsic and intrinsic factors (Figure 2). It is initiated by the expansion of neoplastic cells comprised of CSCs which create the tumor niche. CSCs continuously remodel the TME to maintain an amenable niche. To maintain this architecture, CSCs continuously interact with other TME components including CAFs, immune cells, tumor vasculature, other differentiated cells, and extracellular cues, thus establishing a favorable environment 198, 199. The origin of intra-tumor heterogeneity is based on two different theories. The first, known as the CSC model, states that the cancer “stem cell” is the only tumorigenic fraction capable of indefinite self-renewal and differentiation, thus initiating and maintaining tumor growth. The second, a clonal evolution model, emphasizes that genomic/genetic instability arising from stochastic mutations in individual tumor cells results in a clonal diversity. Thus, as a result of natural selection, clones that acquire an advantageous mutation outgrow those that lack such mutations 200. While ambiguity remains, CSCs are believed to be source of tumor heterogeneity and progression in various cancers 10, 201.

## Cancer associated fibroblasts (CAFs)

Mesenchymal stromal cells (MSCs) are an integral component of the TME and include undifferentiated MSCs along with fibroblasts, pericytes, and vascular or lymphatic endothelial cells 202. MSCs present in solid tumors are called CAFs and are pro-tumorigenic as opposed to the normal fibroblasts which typically suppress tumor formation. The abundance of these stromal cells is correlated with poor prognosis in certain cancers due to increased tissue remodeling through expression of matrix associated proteolytic enzymes, extracellular matrix (ECM) deposition, and dysregulated angiogenesis 199, 203. Moreover, MSCs affect tumor growth by secreting growth factors that bind to surface receptors on tumor cells and pro-angiogenic factors, such as VEGF and PDGF, which promote tumor niche neovascularization. Furthermore, CAFs are involved in the resistance to drug treatment and therapy. For instance, in preclinical models, fibroblast-associated secreted factors, such as WNT-16b, enhanced tumor cell proliferation and their depletion augmented chemotherapy response 204. MSCs are known to be involved in various immunosuppressive mechanisms mediated by metabolites such as indoleamine 2, 3 dioxygenase (IDO), arginase 1 and 2, nitric oxide synthase 2, TGF-β, prostaglandin E_2_, and adenosine. Specifically, high levels of CD73 expressed on the surface of MSCs catalyze the hydrolysis of adenosine monophosphate (AMP). Increased levels of adenosine in the TME lead to activation of the immunosuppressive A2A adenosine receptor on CD8^+^ anti-tumor T cells and NK cells, resulting in a dampened immune response 202, 205, 206. Thus, attempts are being made to relieve the immunosuppression caused by MSCs in addition to other members of the immunosuppressive microenvironment. One such target is fibroblast activation protein (FAP), a type of serine protease that is expressed at abnormally high levels by CAFs. Inhibition of FAP using an anti-tumor vaccine, monoclonal antibody, or chimeric antigen receptor (CAR) T-cell therapy, affects tumor cell growth and increases the CD8^+^ T cell response 207-209. Moreover, the signaling pathways that affect the tumor mesenchyme are being targeted. For instance, tyrosine kinase inhibitors (TKIs) are known to inhibit MSC proliferation and differentiation. Hedgehog signaling inhibition reduces the fibrous tissue in the stroma, thereby increasing vessel formation and facilitating drug delivery. Also, secretion of high amounts of the chemokine CXCL12 results in increased TGF-β secretion through binding to its receptor CXCR4, which in turn causes EMT, a key step in metastasis. Thus, targeting this signaling axis has shown anti-metastatic potential *in vivo* 210-214. However, no clinically relevant data is available for the aforementioned targets and new therapeutic strategies are thus required.

A reciprocal crosstalk exists between CSCs and CAFs in the TME that proves essential for self-renewal of CSCs. CSCs secrete various cytokines and ligands, such as such as hepatocyte growth factor (HGF) and CCL2, to reprogram normal fibroblasts into CAFs, thereby contributing to cancer cell stemness. These factors induce various stemness regulators in CSCs such as Wnt and NOTCH, which contribute to CSC proliferation and self-renewal 201. Further, in lung and breast cancer, the CD10^+^/GPR77^+^ specific population of CAFs has been correlated with poor patient survival. Driven by active NF-κB signaling, these cells secrete IL6 and IL8 to induce cancer stemness 215. Thus, targeting CAFs would indirectly provide the therapeutic benefit of inhibiting CSCs.

## Tumor vasculature

Development of tumor vasculature, or angiogenesis, is primarily driven by the condition of hypoxia 216. Hypoxic cancer cells secrete VEGF-A which binds VEGF receptor 2 (VEGFR2) on the surface of endothelial cells (ECs) of nearby blood vessels and initiates tumor angiogenesis 217. In pre-malignant epithelial tumors, a basal lamina separates the tumor from the surrounding vascular tissue along with angiostatic signals from the ECM and relatively low pro-angiogenic factors, thus preventing the pre-malignant lesions from developing a vasculature. However, during malignant transformation, there is an angiogenic switch wherein pro-angiogenic factors such as growth factors, cytokines, ECM proteins, and ECM remodeling enzymes regulate the vascular ECs to establish an infiltrative and actively growing vascular network 218. Furthermore, the tumor-associated blood vessels develop an aberrant morphology involving excessive branching, abnormal bulges, defective basement membrane, and discontinuous EC lining, thus depicting impaired vascular maturation 219. Poorly organized tumor vasculature results in regions of hypoxia and acidity in the tumor, creating gradients based on the distance from the vascular bed, which in turn affects the distribution and availability of chemotherapeutic drugs to all cancer cells 220. The process of vessel normalization using low dose anti-VEGF therapy can alleviate tumor hypoxic condition and enhance anti-cancer immunity, indicating that the tumor vasculature directly regulates the immune microenvironment 221, 222.

CSCs reciprocally interact with members of the perivascular niche including ECs and ECM components. ECs promote the self-renewal of CSCs through signaling pathways such as Sonic hedgehog, NOTCH, nitric oxide, Jagged-1 223-227, and VEGF-neuropilin 1 (Nrp1, a receptor for VEGF and other molecules) 227. Furthermore the nutrient-rich environment and pro-angiogenic factors such as VEGF and MYC, promote CSC proliferation 201. Reciprocally, CSCs drive the tumor vascularization by stimulating endogenous ECs and creating blood vessel-like structures in melanoma, glioma, breast cancer, and colorectal cancer 228-230. Furthermore, pericytes derived from CSCs modulate the blood-tumor barrier (BTB) by modulating tight junctions. The BTB acts as a barrier for effective drug delivery against GBM. Thus, selective elimination of the CSC-derived pericytes in xenograft murine models disrupt tight junctions of the BTB and increase vesicular transport, thereby enhancing drug effusion into the tumors 92, 231. Therefore, targeting the supportive cross talk of CSCs and the tumor vasculature can be therapeutically valuable.

## Immune cells in the TME

Immune cell infiltration is a complex phenomenon in solid tumors serving pro-tumorigenic functions. Profiles of immune cells substantially differ among various types of cancers. However, the interplay between cancer cells and immune cells remains constant in solid tumors. The tumor infiltrating immune cells are comprised of both lymphoid and myeloid lineages recruited to the tumor from the bone marrow through systemic circulation, which largely consists of various types of leukocytes such as neutrophils, lymphocytes, monocytes, and macrophages and their immature precursors 232. While tumor-associated macrophages (TAMs) form the dominant population of immune cells in different tumor types (up to ~50%), T-cells constitute a very low fraction of tumor infiltrating immune cells 233.

The heterogeneity of TAMs is a major barrier to tumor therapy. Cancer cells recruit TAMs to the TME and reprogram them, thereby using the innate arm of the immune system for their own benefit 234. TAMs are derived from circulating monocytes that arrive at the TME in response to signaling molecules such as chemokines, pro-inflammatory signals, and damage-associated molecule patterns (DAMPs) containing high mobility group box 1 (HMGB1) 235, 236. DAMPs bind to their specific pattern-recognition receptors on macrophages, such as TLR4 for HMGB1, triggering pro-inflammatory signaling 237, typically representing an M1 phenotype. M1 TAMs are classically activated macrophages with an enhanced ability to engulf pathogens, thus possessing anti-tumorigenic properties. However, upon their arrival into the TME, monocytes differentiate and are polarized to an alternatively activated state of macrophage called 'M2'. M2-polarized TAMs have a pro-tumorigenic potential that supports and maintains the CSC population by secreting chemokines and ligands activating cell stemness pathways such as Sonic hedgehog. For example, Milk-fat globule-epidermal growth factor-VIII (MFG-E8) secreted from TAMs activates STAT3 and Sonic hedgehog signaling in CSCs, thus increasing resistance to therapies 238. Furthermore, TAMs secrete higher amounts of TGF-β1 which promotes EMT and CSC properties in many types of cancers 239, 240. Additionally, CSCs reprogram the immune cells in the TME by secreting immunosuppressive proteins such as IL4, which otherwise mitigates the anti-cancer immune response 241. Moreover, GSCs secrete periostin (POSTN) to recruit macrophages/monocytes and accelerate tumor growth; while M2 macrophages physically interact with mouse breast CSCs through ligand-receptor interactions of EphA4-Ephrin and CD90-CD11b 242, 243. Lastly, lung CSCs promote the polarization of myeloid cells to an M2-like phenotype through an IFN-regulated transcription factor IRF5 that is critical for producing macrophage colony-stimulating factor (M-CSF) required for generation of tumorigenic myeloid cells 244.

## Cytotoxic T lymphocytes and their interaction with other members of the TME

Studies from animal models of cancer have shown that CAFs, the tumor vasculature, and TAMs restrict the accumulation of CD8^+^ cytotoxic T lymphocytes (CTLs) in the TME 245. The apoptosis inducer Fas ligand (FasL) is expressed in the vasculature of different types of cancers such as breast, ovarian, bladder, colon, and prostate cancer, but not in the normal vasculature. Cancer cell-derived FasL expression in endothelial cells is associated with scarce CD8^+^ T cell infiltration, while facilitating enhanced infiltration of immunosuppressive regulatory T cells (Tregs) that express high level of apoptosis suppressor, c-FLIP 246. CAF cells with membrane protein fibroblast activation protein-α (FAP), hinder T cell infiltration by producing either a dense collagen matrix or the chemokine CXCL12. Inhibition of CXCR4, a CXCL12 receptor, promotes T cell accumulation and cancer regression 247, 248. TAMs can directly or indirectly inhibit CTLs through different mechanisms. TAMs directly inhibit CTLs through immune checkpoint engagement by expressing programmed cell death ligand 1 (PD-L1) and B7-H4, secreting inhibitory cytokines IL-10 and TGF-β, and depleting metabolites such as L-arginine, which is essential for T cell fitness and anti-tumor activity 249. TAMs also inhibit T cells indirectly by regulating the immune microenvironment. TAMs in human ovarian cancer produce CCL22 to recruit immunosuppressive Treg cells which suppress CTLs, stimulate TAMs to produce immunosuppressive cytokines IL-6 and IL-10, and enhance B7-H4 expression, which in turn suppresses IL-2 production and T cell proliferation 249. Furthermore, inhibition of the CSF1 receptor (CSF1R) pathway or CC-chemokine receptor 2 (CCR2), attenuates macrophage recruitment and enhances T cell infiltration in the tumor. CSF1R inhibition suppresses murine glioma. However, clinical trials of CSF1R inhibition have failed to increase overall survival for patients with gliomas 250, 251. Additionally, TAMs restrict the intra-tumoral localization of T cells by producing reactive nitrogen species, increasing fibrosis, and inducing TGF-β signaling 249. TGF-β produced by CSCs inhibits the proliferation of active T cells while inducing immunosuppressive Treg cells through both Foxp3-dependent and independent pathways 252, 253.

CD8^+^ cytotoxic T lymphocytes are the key players involved in killing cancer cells. For this reason, T cells need to sufficiently accumulate in the TME, efficiently infiltrate, and physically contact CSCs. Furthermore, T cells should adequately respond to tumor antigens and activation signals from cells. However, T cells become dysfunctional after arriving at the tumor milieu. This acquired dysfunction of T cells is due to the induction of multiple inhibitory receptors (IRs), including cytotoxic T lymphocyte antigen 4 (CTLA-4), programmed cell death 1 (PD-1), T-cell immunoglobulin domain and mucin domain-3 (Tim-3), T cell immunoreceptor with Ig and ITIM domains (TIGIT), lymphocyte activation gene 3 (LAG-3) and others, wherein the severity of dysfunction depends on the type and number of IR co-expression. These IRs bind to their respective ligands, which are typically expressed on antigen presenting cells or tumor cells. These ligands then deliver an inhibitory signal to T cells attenuating their proliferation and effector functions 254. Several other factors cause the dysfunction of T cells in the TME including presence of inhibitory cells, suppressive soluble mediators, metabolic pathways, and epigenetic and transcriptional regulation which also cause T cell dysfunction in the TME 254 (Figure 2). Various treatment strategies are aimed at inhibiting the repression on T cell activity in the TME 255. The key function of T cells in tumor immunology is demonstrated by a positive correlation between T cell infiltration and better clinical outcome as described in melanoma, colorectal, and breast cancers 256. Cytotoxic T cell activity in tumors is inhibited directly by CSCs through engagement of IRs such as PD-1 and CTLA-4 on T cells, and by up-regulation of their ligands on antigen-presenting cells 257. Checkpoint inhibitor drugs which target CTLA-4, PD-1, and its ligand PD-L1 have been successful in clinical trials for treating metastatic melanoma, non-small cell lung cancer, and renal cancer, among others. However, mixed results are emerging for the expression of PD-L1 on CSCs, with high expression in CSCs in head and neck, breast, and colon cancers, and low or undetectable expression in other types of cancers 201. In low-grade glioma, GBM, prostate adenocarcinoma, and lung squamous cell carcinoma, stemness features identified from TCGA datasets correlated negatively with PD-L1 expression 201. Thus, checkpoint inhibitors might be less efficient for targeting CSCs and investigation of other immune invasive mechanisms is required.

## CSC in therapy resistance

Resistance to broad chemotherapeutic agents and selective targeted therapies is a leading cause of cancer deaths 14. Cells that survive treatment are able to expand and can lead to recurrence of disease or metastatic spread, both of which dramatically decrease patient overall survival 258, 259. CSCs have been suggested to be the major part of this therapy-resistant cell population within the tumor due to their defined phenotype of quiescence, EMT, multi-drug resistance (MDR), and resistance to DNA damage-induced apoptosis 260, 261.

## Quiescence

CSCs have been thought to exist in a dormant state, or G0 phase, after tumors have formed 262. A cell lineage study showed that serial xenograft passages of colorectal cancer cells in mice gave rise to distinct subpopulations of cells whereas chemotherapy largely eliminated the rapidly proliferating clones, but enhanced the dominance of the dormant clones 184. This depicts the challenge of eradicating a dormant cell with common chemotherapeutic agents that target rapidly dividing cells. In a mouse glioma model, a *Nestin-∆TK-IRES-GFP* transgenic mouse was created to label quiescent stem cells and glioma tumor cells. In these animals, temozolomide (TMZ) treatment efficiently ablated dividing cells, but increased the expansion of the GFP-labeled quiescent cells 263. In a bladder cancer model, combination of gemcitabine and cisplatin also induced rapid cell division of previously quiescent cells to repopulate the tumor in a way similar to that of normal stem cells, post wound formation 184.

Nevertheless, research is being conducted to specifically target these quiescent CSCs. Genetic ablation of the F-box protein Fbxw7 induced LSC proliferation, which could then be successfully targeted by imatinib 264. Quiescent cells have also shown a dependence on autophagy in colorectal 265, liver 156, brain 266, and melanoma 267 CSCs. Therefore, recent studies targeting both autophagy 268 and the overarching metabolic state have shown pre-clinical promise 105. Moreover, the opposing idea of maintaining cells in a quiescent state has been tested as well. A recent study shows that retinoic acid and NR2F1 signaling work together to induce a dormancy-like epigenetic state 269. Fenretinide treatment in lung and colorectal cancers also induced a quiescent state along with inhibition of the mTOR pathway, cell cycle block, and a mixed death pathway with both autophagic and apoptotic qualities 270.

## Epithelial-to-mesenchymal transition (EMT)

CSCs express many of the markers of normal stem cells, including their ability to survive in a de-differentiated state 271. Experimental evidence shows that induction of EMT or de-differentiation of immortalized human mammary epithelial cells leads to an increase in their local invasion and metastatic burden 272, 273. Further characterization of breast epithelial cells that have been induced to undergo EMT mimic characteristics of mesenchymal stem cells. Specifically, they show enhanced wound-homing abilities and are able to differentiate into multiple distinct lineages 274. There is also a relationship between a more EMT-like phenotype and multi-drug resistance 275. Markers of stemness, like OCT4, NANOG, and SOX2, have been associated with resistance to chemotherapy 276-279. Another example is ZEB1, a transcription factor associated with EMT. ZEB1 is able to regulate both self-renewal and therapy resistance in GSCs through regulation of O-6-methylguanine methyltransferase (MGMT) via miR-200c and c-MYB 280. Additionally, cancer cells that undergo EMT can go into a dormant state. The Wnt pathway also plays a dominant role in both EMT and stemness since Wnt-1 stimulation induces EMT in breast cancer 281, 282. In lung cancer cells, gefitinib treatment activates NOTCH-1 signaling, which allows for an acquired EMT phenotype and resistance to the therapy 283. Among a list of other factors including those of invasive potential and tumorigenicity, EMT markers have often been used to predict resistance to the anti-EGFR antibody cetuximab in urothelial carcinoma cells 284.

As EMT is an important feature of stem cells, and a pivotal stage in metastasis, inhibitors of EMT have been developed and exploited 285. TGF-β signaling is one of the major pathways associated with EMT, as one and a half days of TGF-β treatment induced the reprogramming of mouse embryonic fibroblasts (MEFs) into iPSCs 286. For this reason, inhibitors targeting TGF-β signaling have been developed and are currently undergoing clinical trials 287. Lastly, natural compounds have also been tested to inhibit EMT. Curcumin, an abundant compound in turmeric and an HGF inhibitor, induced the epithelial marker E-cadherin and inhibited tumor growth 287.

## Multi-drug resistance

Aldehyde dehydrogenase (ALDH) is a potential selective marker for CSCs in breast, bladder, embryonal rhabdomyosarcoma, head and neck squamous cell carcinoma, and lung cancer 288, 289. As a cytosolic enzyme that oxidizes intracellular aldehydes, ALDH protects cells from elevated reactive oxygen species (ROS) levels. Decreasing or maintaining a low ROS level is critical to a cell, as ROS accumulation causes cell death 290. Higher expression of ALDH, as compared to ALDH-negative lung cancer cells, has been shown to confer resistance to multiple chemotherapeutic agents like cisplatin, etoposide, fluorouracil, and gefitinib 291. ALDH^+^ pancreatic CSCs also showed therapy resistance, but also an increase in phosphorylated STAT3 which could be targeted with STAT3 inhibitors 292. In ovarian CSCs, ALDH1A2 was shown to be directly regulated by NF-κB signaling via the transcription factor RelB. Loss of RelB inhibited CSC spheroid formation, ALDH expression, tumorigenesis, and chemoresistance 293. As expected, an inhibitor of ALDH, diethylaminobenzaldehyde, has been shown to re-sensitize breast CSCs to chemotherapy 294.

The ATP-binding cassette (ABC) transporter family has been implicated in the multi-drug resistance of CSCs. This family is made up of 49 family members that have 7 gene sub-families 295. The major function of ABC transporters is to pump substrate proteins and drugs across the plasma membrane powered by ATP hydrolysis 296. However, considerable numbers of ABC transporters are often overexpressed in cancers, particularly in CSCs 297. Moreover, OCT4 has been shown to control the gene expression of multiple ABC proteins 298. Many oncogenes have been implicated in regulating ABC functions as well. For examples, MYC plays a dual role in ABC expression. MYC activation can increase the expression of ABCC1 and ABCC4, while attenuating levels of ABCC3 299. The PI3K/AKT pathway, but not mTOR, regulates ABCG2 in GSCs via its localization to the plasma membrane. This phenotype is exaggerated with PTEN loss and TMZ treatment, which is representative of clinical presentation and treatment of gliomas 300. Inhibition of BCR-ABL and its downstream target pathway PI3K/AKT can also lead to downregulation of ABCG2 in CML cells 301. Treatment of breast cancer cells with an EGFR/HER2 inhibitor lapatinib, resulted in a decrease in ABCB1 and ABCG2 expression, thereby sensitizing breast cancer cells to a chemotherapeutic agent doxorubicin 302. Although ABC transporters have been implicated in CSC resistance to therapies, therapeutically targeting ABC transporters will target normal stem cells and brain ECs that make up the blood brain barrier 229. Therefore, identification of CSC-specific ABC transporters is necessary. One example is the breast cancer resistance protein (BCRP), which is overexpressed in mitoxantrone-resistant cancer cells. Treatment with fumitremorgin C (FTC) efficiently reverses drug resistance in these mitoxantrone-resistant breast cancer cells and in other types of cancer cells transfected with exogenous BCRP 303.

## Resistance to DNA damage-induced death

CSCs have an altered DNA damage response (DDR) and repair pathways which closely mimic that of normal stem cells 304. As normal stem cells are needed to repopulate normal tissue post-damage, their DDR must be error-free to preserve normal tissue DNA 305. In CSCs, this efficient DDR leads to radio- and chemoresistance 272. In GSCs, multiple, sometimes contradicting, studies have described CD133^+^ progenitor cells and DNA repair. CD133^+^ cells were shown to efficiently repair DNA damage 306. However, changes in γ-H2AX foci resolution, DNA base excision, or single-strand break repair in radiation-treated GSCs were not found 307. However, CD133^+^ GSCs enriched for the polycomb group protein BMI1 have increased radio-resistance, in which BMI1 recruits both double-stranded break repair and nonhomologous end joining proteins 308. CD133^+^ GSCs also had an increase in Chk 1-dependent DNA repair response, and increased expression of the Mre11, Rad50, and Nbs1 (MRN) complex component NBS1 309. CD133^+^ CSCs in breast, lung, and non-small-cell lung cancers also showed an increase in DNA damage response and repair genes 310. The homologous repair (HR) pathway of DNA damage is vitally important to CSCs, as this is less error-prone process for DNA repair. HR takes place in S phase, when a homologous chromosome template is present 310. GSCs have been shown to overexpress RAD51, the major HR DNA repair protein 311. In head and neck squamous cell carcinoma, overexpression of Fanconi anemia DNA repair proteins is observed specifically in ALDH1^+^ cells 312. Genes inducing cell death via DDR pathway such as p53 are often dysregulated in CSCs. p53 acts as a sensor when detrimental DNA damage has occurred. However, p53 is frequently mutated or downregulated in CSCs. Therefore, restoration of normal p53 function in p53-mutated GSCs may provide a new treatment avenue 313.

## Therapies targeting CSC

The promise of the CSC hypothesis is that an in-depth understanding of CSC biology will allow us to develop more effective approaches to eradicate CSCs in patients. Although the inherent plasticity of CSCs presents major challenges for design of anti-CSC therapies, some strategies have been tested to interfere with CSCs in preclinical models and patients, including inhibition of key CSC signaling pathways, viral therapy, awakening quiescent CSCs, and immunotherapy.

## Targeting of key CSC signaling pathways

The lysine-specific demethylase (LSD1), also known as lysine-specific histone demethylase 1A (KDM1A), is a histone demethylase that demethylates H3K4me1/2 and has been extensively studied 314. LSD1 represents an attractive therapeutic target for LSCs, because LSD1 plays a critical role in maintenance of LSCs. Tranylcypromine analogs have been used for LSD1 inhibition and induce CSC differentiation and suppress LSC expansion and acute leukemia development without any notable side-effects 315. Several LSD1 inhibitors are currently in phase I/II clinical trials in AML patients 316. Accumulating evidence indicates that autophagy is implicated in CSC maintenance and therapy resistance 317. Suppression of autophagy also holds promise for the therapeutic elimination of CSCs. In GBM, targeting a key autophagy regulator, ATG4B, with a small molecule inhibitor, NSC185058, enhanced the efficacy of radiation therapy in orthotopic xenograft models 268. Additionally, targeting BMI1 by using a small molecule, PTC-209, has also demonstrated efficacy against CSCs in models of colorectal cancer through decreasing BMI1 protein levels 318.

## Viral therapy

With the success of modern immunotherapy, oncolytic viral therapy has become a novel and promising strategy to target cancers and CSCs. Oncolytic viruses can effectively replicate within cancer cells rather than in normal cells, resulting in lysis of the tumor mass 319. In addition to this primary effect, oncolytic viruses are also able to stimulate the immune system against cancer cells, in which the immune system is silenced by the TME 320. The use of improved oncolytic adenovirus treatment regimens has demonstrated therapeutic activity in immunocompetent C57BL/6 mouse models by stimulating an influx of CD8^+^ T cells specific to tumor-associated antigens 321, 322. Moreover, in human patients, viral treatments have been shown to induce a tumor macrophage phenotypic shift 323. Oncolytic viruses also represent attractive combination strategies with PD-1/PD-L1 blockade therapy, and initial clinical studies have suggested promising results 324. Phase I trials of intratumoral inoculation of the recombinant nonpathogenic polio-rhinovirus chimera (PVSRIPO) have demonstrated some efficacy in the treatment of patients with recurrent GBM, with evidence that the survival rate among patients treated with PVSRIPO immunotherapy gain relatively long-term survival benefit 325. The highly neurotropic flavivirus Zika virus expressing an exogenous Endostatin‐Angiostatin fusion gene (VAE) can infect and suppress GSCs in organoid and mouse models, indicating that VAE-based gene oncolytic viral therapy is a promising strategy for the treatment of brain tumors 326. Similarly, oncolytic retroviruses have the potential to inhibit the growth of CSC xenograft tumors 327. Although several clinical trials performed in a small population of breast cancer patients have demonstrated oncolytic reovirus safety 328-330, further investigations with a heterogeneous population are necessary for reliable prediction of oncolytic viral therapy in cancer patients.

## Awakening quiescent CSCs

The presence of CSCs is a well-recognized concept, wherein poorly differentiated and quiescent cells within a tumor mass are thought to be the major cause of chemotherapy resistance. Thus, targeting quiescent CSCs is emerging as a viable treatment option 261. Genetic ablation of the ubiquitin ligase FBXW7, a negative regulator of MYC, forces quiescent LSCs to re-enter the cell cycle and increases their sensitivity to the tyrosine kinase inhibitor (TKI) imatinib 331. CSCs can also reprogram metabolic pathways to modulate cancer growth. It has been demonstrated that CSCs can switch between oxidative phosphorylation and glycolysis even in the presence of oxygen to support tumor growth 332. Several studies have shown that quiescent CSCs are dependent on oxidative metabolism. Therefore, suppression of oxidative phosphorylation reduces the ability of self-renewal and tumor initiation of CSCs and enhances the response to targeted therapies in preclinical mouse cancer models 168, 170, 172, 333.

## Immunotherapy

Immunotherapy has gained significant attention for its potential to treat and cure various types of cancers and represents an alternative option to target CSCs. Given that the combination of surface expression markers that are used to identify the CSC populations in different tumor types, CSC markers are therefore an attractive target for cancer therapeutics 334. For example, transmembrane glycoprotein CD44 is overexpressed in CSCs of various cancer types, including breast, prostate, bladder, gastric cancer, and others 335. A novel therapeutic strategy employing near-infrared photo immunotherapy (NIR-PIT) targeting CD133 in GBM has also been shown to be highly specific and efficient for eliminating GSCs 336. The monoclonal antibody (mAb) H90 was first shown to efficiently eradicate CD44^+^ human AML CSCs 337. Additionally, anti-tumor vaccines could be developed to target tumor-associated antigens and elicit tumor-specific T cell responses. In two early clinical trials, administration of personalized vaccines containing tumor neoepitopes elicited sustained responses of central memory T cells with evidence of immunologic memory and tumor-infiltrating capacity 338, 339. T cell receptor (TCR) gene therapy is another kind of adoptive cellular immunotherapy where patient-derived T cells are engineered to produce a CAR selective for a tumor antigen, with subsequent *ex vivo* cell expansion and adoptive transfer back into the patient. CAR-T cell therapy against tumor-specific targets in CSCs, including CD33 for AML 340 and EGFRvIII for GBM 341, displayed some efficacy in treating patients in phase I clinical trials. The association of increased expression of immune checkpoint ligands with CSC function inspired investigators to use inhibitors of these checkpoint molecules to target CSCs for cancer treatments. Moreover, a preclinical study showed that combined inhibition of CTLA-4, PD-L1, and a CSC vaccine remarkably suppressed tumor growth in multiple mouse models 342. Therefore, targeting CSCs with the immune system for treating cancers remains an intriguing and fruitful field of ongoing investigation.

## Conclusions and future perspectives

Decades of accumulating evidence shows the significant progress that has been made in our understanding of CSCs at the transcriptional, posttranscriptional, epigenetic, metabolic, and microenvironmental regulatory levels. These findings have been driven by the implementation of new technologies, such as single cell multi-omics technology, and have provided further insight into our understanding of the evolution and heterogeneity of human cancers, as well as the TME 343. The advent of CRISPR-Cas9 screening technologies has also greatly accelerated cancer research in many aspects, including robust site-specific gene editing, generation of animal cancer models, and functional genetic screening. The potential role of CRISPR-Cas9-based gene editing has received a lot of attention and has become a critical tool in the development of cancer therapeutics 344, 345. The role of the TME as a critical regulator of CSC properties and an essential target for CSC elimination has now come into sharper focus. With the advances in the immunotherapy and TME fields that have been made in recent years, we can also expect to see big landmark developments of more efficient approaches to eliminate these highly tumorigenic and therapy-resistant cells.

Despite the optimism resulting from these marked advances, intense ongoing research is attempting to address many of the remaining unanswered questions. In this review, we focused on several outstanding challenges, which include approaches for investigating and the therapeutic targets of CSCs. First, a large proportion of cancer research continues to use *in vitro* sphere formation in serum-free medium as a surrogate CSC assay. These studies are frequently performed in hypoxic, hyperglycemic, and non-physiologic conditions. In addition, the *in vitro* culture conditions used for CSCs may not include key factors that are essential for the *in vivo* fundamental properties of most CSCs. Therefore, these experiments likely fail to recapitulate the clinical presentation seen in human cancers, illustrating the need for direct CSC detection approaches that better mimic physiological conditions. Recently, organoid model systems and other tools have gained appreciation in CSC research and their use in high-throughput and high-fidelity tumor modeling may be beneficial in overcoming these challenges 346. Second, immunodeficient mouse models have been used effectively to identify the capacity of CSCs to reconstruct tumor heterogeneity that resembles the parent tumor *in vivo*. This powerful strategy is very useful for detection and quantification of CSCs in various types of human cancers. However, a major shortcoming of the immunodeficient mouse model is the lack of an intact TME. This is an important consideration, given that components of TME have an important role in supporting tumor generation, progression, and therapeutic response 347. Human-severe combined immunodeficient (SCID) mouse chimeric models have been developed for the engraftment of human tumor cells that recapitulate the native tumor microenvironment, allowing researchers to evaluate the role of microenvironment components, such as tumor-infiltrating leukocytes and other human stromal cells 348. Third, increasing evidence has revealed that both CSCs and non-CSCs represent a very plastic and dynamic population, which is capable of changing cell types in response to certain environmental stimuli. This is exemplified by a study in which subpopulations of cells purified for a given phenotypic state (i.e. stem cell, basal-, or luminal-like phenotypes) have equal tumorigenic potency. Each subpopulation of cancer cells effectively initiated tumor growth in mouse models, and eventually returned to equilibrium proportions with sufficient time 23. Given that the plasticity of CSCs may present major challenges in the development of efficient therapies, it is essential to improve our insights into the molecular mechanisms underlying tumor cell plasticity and develop more effective therapies to target these cells.

Finally, despite the intense focus on uncovering important molecular targets as potential strategies for therapeutic intervention of CSC function, very few targets have been effectively translated into clinical care, and the failure rate of clinical trials remains high. It is likely that inefficient drug delivery and drug intervention in advanced stages of disease may be impeding their impact on tumor growth. Improving approaches of delivery through advanced biomaterials and drug delivery systems represent another important space to explore and may improve the efficacy of targeted therapies for cancer treatments.

## Figures and Tables

**Table 1:** Key transcriptional factors of CSCs

Transcription factor	Cancer type	Effects	Refs
OCT4	Pancreatic	Chemoresistance, and tumorigenesis	38-41
SOX2	Glioma, breast and others	Self-renewal, tumor growth and therapy resistance	44, 45
KLF4	Breast, glioma, and osteosarcoma	Metastasis, migration and drug resistance	50-52
MYC	Glioma, breast	Drug resistance	54, 55
NANOG	Hepatic, prostate, colorectal, and brain cancers	Tumorigenesis and therapy resistance	56, 62, 63
Wnt/TCF	Breast and glioma	Metastasis and stemness maintenance	66, 67
STAT3	Breast, liver, colon leukemia and prostate	Proliferation, stemness maintenance and immunosuppressive	69-75
NF-κB	Breast, prostate, ovarian and pancreatic cancer	Pro-inflammatory, angiogenesis and invasion	76-80

## Abbreviations

ABC: ATP-binding cassette
ADAR: adenosine Deaminases acting on double-stranded RNA
ALDH: aldehyde dehydrogenase
AML: acute myeloid leukemia
CAF: cancer associated fibroblast
CAR: chimeric antigen receptor
CML: chronic myeloid leukemia
CSC: cancer stem cell
CTL: cytotoxic T lymphocyte
CTLA-4: cytotoxic T-lymphocyte-associated antigen-4
DAMP: damage-associated molecule pattern
DDR: DNA damage response
DIPG: diffuse intrinsic pontine glioma
DNMT: DNA methyltransferase
EC: endothelial cell
ECM: extracellular matrix
EGFR: epidermal growth factor receptor
EMT: epithelial-mesenchymal transition
FTO: fat mass and obesity-associated protein
GBM: glioblastoma
GSC: glioma stem like cell
G-CIMP: glioma-CpG island methylator phenotype
H3K27me3: histone H3 trimethylated lysine 27
HSC: hematopoietic stem cell
HIF-1α: hypoxia inducible factor 1 alpha
ITGB4: Integrin β4
IDH: isocitrate dehydrogenase
IR: inhibitory receptor
iPSCs: induced pluripotent stem cells
JAK: Janus kinase
KLF4: Kruppel-like factor 4
LncRNA: long noncoding RNA
LSC: leukemia stem cell
LSD1: lysine-specific demethylase
m^6^A: N^6^-methyladenosine
MAPK: mitogen-activated protein kinase
MDR: multi-drug resistance
MEF: mouse embryonic fibroblast
MGMT: O-6-methylguanine Methyltransferase
miRNA: microRNA
Mesenchymal: MES
MSC: mesenchymal stromal cell
OCT4: octamer-binding transcription factor 4
OXPHOS: oxidative phosphorylation
PD-1: programmed death- 1
PD-L1: programmed cell death ligand 1
PRC2: polycomb repressive complex 2
Proneural: PN
RTK: receptor tyrosine kinase
ROS: reactive oxygen species
SCID: severe combined immunodeficient
STAT3: signal transducer and activator of transcription 3
SOX2: Sry-related HMG box 2
TAM: tumor associated macrophage
TCR: T cell receptor
TET: methylcytosine dioxygenase
TF: transcription factor
TKI: tyrosine kinase inhibitor
TME: tumor microenvironment
TMZ: temozolomide
VEGF: vascular endothelial growth factor
WT: wild-type
